# Surgical treatment of left-sided infective endocarditis with symptomatic neurological complications before surgery in China

**DOI:** 10.3389/fcvm.2023.1217148

**Published:** 2023-09-06

**Authors:** Jing-bin Huang, Chang-chao Lu, Zhao-ke Wen, Jian-rong Yang, Jun-jun Li

**Affiliations:** Department of Cardiothoracic Surgery, The People’s Hospital of Guangxi Zhuang Autonomous Region, Guangxi Academy of Medical Sciences, Nanning, China

**Keywords:** left-sided infective endocarditis, surgery, neurological complications before surgery, mortality, prolonged intubation time

## Abstract

**Introduction:**

We aimed to investigate surgical treatment of left-sided infective endocarditis with symptomatic neurological complications before surgery.

**Methods:**

This was a retrospective study of patients with left-sided infective endocarditis and symptomatic neurological complications before surgery undergoing cardiac surgery between January 2006 and November 2022 at our hospital.

**Results:**

Eight hundred thirty-two patients were divided into group with symptomatic neurological complications before surgery (*n* = 112) and without symptomatic neurological complications before surgery (*n* = 720). There were 48 operative deaths (5.4%). Univariate and multivariate analyses showed that symptomatic neurological complications before surgery is statistically significantly associated with in-hospital mortality following cardiac surgery and prolonged intubation time.

**Conclusions:**

Our study showed that symptomatic neurological complications before surgery are associated with increased in-hospital mortality following cardiac surgery and prolonged intubation time.

## 1. Introduction

Infective endocarditis (IE) is an infectious disease associated with high morbidity and mortality. It is one of the most common life-threatening infections, occurring more frequently in older patients and those with prosthetic valves. Without treatment, IE is almost uniformly fatal. Even at experienced centers, operations for IE remain associated with the highest mortality of any valve disease (1, 2). Infective endocarditis patients with neurological complications have a significantly higher risk of mortality than patients without neurological complications. Surgery is an effective treatment in patients presenting with infective endocarditis and may be undertaken in patients with neurological complications to prevent poorer prognosis. Surgical treatment is particularly effective in selected IE patients presenting with neurological complications to prevent additional neurological sequelae and mortality. However, the decision for surgery remains controversial and should be decided carefully with a multidisciplinary team as patient prognosis may worsen with surgical intervention (3, 4).

We aimed to investigate the surgical treatment of left-sided infective endocarditis with symptomatic neurological complications before surgery. We hypothesized that symptomatic neurological complications before surgery are associated with increased in-hospital mortality following cardiac surgery and prolonged intubation time.

## 2. Patients and methods

### 2.1. Design

This was a retrospective study of patients with left-sided infective endocarditis infective endocarditis between January 2006 and November 2022 at our hospital. Demographic and outcome data were collected using a hospital database. Medical records were reviewed.

### 2.2. Eligibility criteria

#### 2.2.1. Inclusion criteria

Patients with left-sided infective endocarditis between January 2006 and November 2022 at our hospital were included.

#### 2.2.2. Exclusion criteria

Patients with right-sided infective endocarditis were excluded.

### 2.3. Variables to be analyzed

Variables were evaluated (Supplementary Data).

Postoperative LVEDD was measured by transthoracic echocardiography postoperatively 1–7 days in intensive care unit.

Perioperative death was defined as death within 30 days of the operation or during the same hospital admission.

In our study, serum creatinine was used as the diagnostic standard of acute renal injury. According to Kidney Disease Improving Global Outcomes (KDIGO) classification, if serum creatinine increases by ≥0.3 mg/dl (26.5 μmol/L) within 48 h, serum creatinine is 50% higher than the baseline within first 7 days, or urine output is below 0.5 ml/kg/h for 6 h, the patient is considered to have acute renal injury (5).

Multiorgan failure (MOF) is regarded as a continuous process of varying levels of organ failure rather than an all-or-none event. To characterize MOF, six different organ systems are regarded as “key organs”: lungs, cardiovascular system, kidneys, liver, coagulation system, and central nervous system (6).

Hepatic failure is defined as a severe liver injury, potentially reversible in nature and with onset of hepatic encephalopathy within 8 weeks of the first symptoms in the absence of pre-existing liver disease (7).

Respiratory failure is a condition in which the respiratory system fails in one or both of its gas exchange functions, i.e., oxygenation of and/or elimination of carbon dioxide from mixed venous blood. It is deﬁned by an arterial oxygen tension (Pa, O_2_) of ≤8.0 kPa (60 mmHg), an arterial carbon dioxide tension (Pa, CO_2_) of ≥6.0 kPa (45 mmHg) or both (8).

### 2.4. Diagnosis of infective endocarditis

All our clinical data were obtained from patient records and our institutional database. Diagnosis of IE is based on clinical symptoms, physical findings, microbiology results, echocardiography, and other studies. Duke or modified Duke criteria are used to classify certainty of the diagnosis (9). Transthoracic or transesophageal echocardiography was done to measure and classify vegetations. Blood cultures and full body computed tomography scans were routinely done. Once the diagnosis was confirmed, empiric antibiotic therapy was started immediately. Patients’ indication for surgery was according to the AATS/ESC guidelines for the management of IE.

All patients suspected by a clinician to have neurological complications were evaluated by a neurologist. Patients with neurological deficit were considered as symptomatic and included in the symptomatic neurological complications group. Asymptomatic patients with or without neurological abnormalities on MRI or CT were included in the non-neurological complications group. Patients with symptomatic neurological complications all underwent brain CT, and repeat brain CT all were done for those scheduled for surgery. Surgical and pathological findings were reviewed to confirm the preoperative diagnosis.

### 2.5. Follow-up

All survivors discharged from hospital were monitored until the end study date or a known date of death. At the outpatient department, all patients were investigated with echocardiogram, electrocardiogram, and x-ray chest film, once every 3–12 months. At the last follow-up, the patients were contacted by telephone or micro-message or interviewed directly at the outpatient department.

### 2.6. Statistical analyses

Continuous variables are reported as means ± SE. Survival rates were estimated using the Kaplan-Meier method. The *χ*^2^-test, the Kruskal-Walls test, or the Wilcoxon rank-sum test, as appropriate, was used to evaluate relationships between the preoperative variables and selected intraoperative and postoperative variables. The relationships with perioperative risk factors were assessed by means of contingency table methods and logistic regression analysis. *P* values less than 0.05 were statistically significant. All analyses were performed using IBM SPSS version 24.0 software (IBM SPSS Inc., USA).

## 3. Results

### 3.1. Characteristics of the population under study

**Figure 1:**
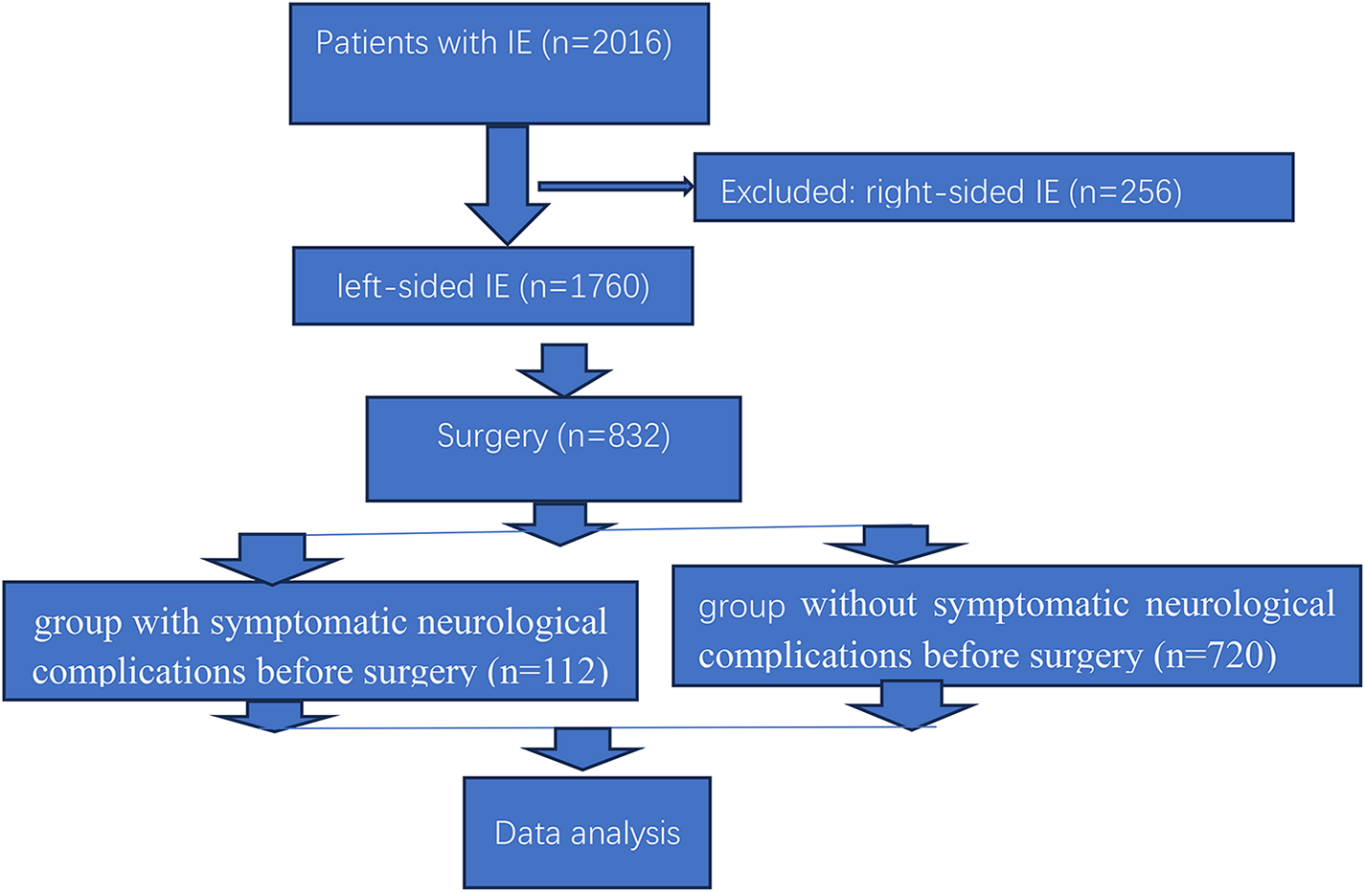
Flow chart of clinical trial.

During the study period, 2016 patients were diagnosed as infective endocarditis, 1,760 (87.3%, 1,760/2,016) left-sided infective endocarditis, 432 (24.5%, 432/1,760) patients with symptomatic neurological complications, including 240 (13.6%, 240/1,760) patients with cerebral embolism, 128 (7.3%, 128/1,760) with intracerebral hemorrhage, 33 (1.9%, 33/1,760) with cerebral embolism and intracerebral hemorrhage, 15 (0.8%, 15/1,760) with meningitis and brain abscess, 16 (0.9%, 16/1,760) with cerebral embolism and meningitis (Table 1). Only 25.9% of patients (112/432) with symptomatic neurological complications are indicated for surgery at admission, other patient (74.1%, 320/432) were contraindicated for surgery for death, severe septic shock, or stroke and coma or extensive neurologic deficit. Figure 1 showed the flow chart of clinical trial.

**Table 1:** Characteristics of the patients with left-sided infective endocarditis (*n* = 1,760).

Variable	Total (*n* = 1,760)	Group with symptomatic neurological complications (*n* = 432)	Group without symptomatic neurological complications (*n* = 1,328)	*P* value
Male, *n* (%)	1,264 (71.8%)	304 (70.4%)	960 (72.3%)	0.441
Age, years	43.04 ± 0.40	41.48 ± 0.64	43.54 ± 0.49	0.028
Weights, kg	55.93 ± 0.29	58.91 ± 0.56	54.96 ± 0.33	<0.001
Time between symptoms and admission, months	2.18 ± 0.05	1.95 ± 0.08	2.25 ± 0.06	0.007
Vegetation length, mm	11.05 ± 0.15	12.98 ± 0.28	10.42 ± 0.17	<0.001

The neurological event was the first sign of IE in 140 patients (8.0%, 140/1,760), occurring before the start of antimicrobial treatment, and the complication occurred during the first week of antimicrobial treatment in 252 patients (14.3%, 252/1,760).

### 3.2. Analysis of risk factors of symptomatic neurological complications in left-sided infective endocarditis (*n* = 1,760)

Univariate analysis showed that vegetation length (*P* < 0.001), aortic involvement (*P* < 0.001), and mitral involvement (*P* = 0.001) are associated with symptomatic neurological complications in infective endocarditis.

When they were included in multivariate analysis models, multivariate analyses also showed that vegetation length (*P* < 0.001), aortic involvement (*P* = 0.004), and mitral involvement (*P* < 0.001) are associated with symptomatic neurological complications in infective endocarditis (Table 2).

**Table 2:** Analysis of risk factors of symptomatic neurological complications in left-sided infective endocarditis (*n* = 1,760).

Model	OR	95% CI	*P* value
Univariate analysis of risk factors of symptomatic neurological complications in infective endocarditis			
Vegetation length	0.935	0.919–0.952	<0.001
Aortic involvement	0.547	0.438–0.684	<0.001
Mitral involvement	2.395	1.402–4.091	0.001
Multivariate analysis of risk factors of symptomatic neurological complications in infective endocarditis			
Vegetation length	0.932	0.915–0.949	<0.001
Aortic involvement	0.455	1.305–3.907	0.004
Mitral involvement	2.258	0.436–0.752	<0.001

### 3.3. Operative data

Of the 1,760 patients with left-sided infective endocarditis, 832 (47.3%, 832/1,760) underwent cardiac surgery, and 112 patients with infective endocarditis and symptomatic neurological complications preoperative undergoing cardiac surgery were further investigated. 832 patients with left-sided infective endocarditis undergoing cardiac surgery were further divided into group with symptomatic neurological complications before surgery (*n* = 112) and without symptomatic neurological complications before surgery (*n* = 720). The interval between decision for surgery and operation was 1.58 ± 0.11 (range, 0.2–4.0) months. The in-hospital mortality in our study was 5.8% (48/832), mean age at operation was 39.23 ± 0.5 years. Age at operation in our cohort was younger than that in high-income countries (Table 3).

**Table 3:** Characteristics of the operative patients (*n* = 832).

Variable	Total (*n* = 832)	Group with symptomatic neurological complications before surgery (*n* = 112)	Group without symptomatic neurological complications before surgery (*n* = 720)	*P* value
Male, *n* (%)	528 (63.5%)	64 (57.1%)	464 (64.4%)	0.135
Age, years	39.23 ± 0.50	38.43 ± 1.28	39.36 ± 0.55	0.529
Weight s, kg	55.08 ± 0.42	52.93 ± 1.47	55.41 ± 0.43	0.045
Time between symptoms and surgery, months	2.55 ± 0.08	1.93 ± 0.17	2.64 ± 0.09	0.002
NYHA class				
II, *n*	491 (59.0%)	64 (57.1%)	427 (59.3%)	0.665
III, *n*	230 (27.6%)	30 (26.8%)	200 (27.8%)	0.827
IV, *n*	111 (13.3%)	18 (16.1%)	93 (12.9%)	0.361
Comorbidities				
Coronary heart disease, *n*	18 (2.2%)	4 (3.6%)	14 (1.9%)	0.271
Hypertension, *n*	36 (4.3%)	8 (7.1%)	28 (3.9%)	0.115
Diabetes mellitus, *n*	17 (2.0%)	3 (2.7%)	14 (1.9%)	0.609
Vegetation length, mm	10.98 ± 0.22	11.71 ± 0.33	10.86 ± 0.25	0.181
Preoperative LVEDD, mm	62.43 ± 0.28	62.14 ± 0.89	62.48 ± 0.30	0.685
Preoperative LVEF, %	61.84 ± 0.3	62.1 ± 1.0	61.8 ± 0.3	0.665
Preoperative aortic insufficiency, cm^2^	5.93 ± 0.24	3.61 ± 0.56	6.30 ± 0.26	<0.001
Preoperative mitral insufficiency, cm^2^	7.83 ± 0.21	9.93 ± 0.61	7.50 ± 0.22	<0.001
Preoperative tricuspid insufficiency, cm^2^	3.83 ± 0.13	5.37 ± 0.33	3.59 ± 0.14	<0.001
Native valve IE	736 (88.5%)	96 (85.7%)	640 (88.9%)	0.328
Prosthetic valve IE	96 (11.5%)	11 (9.8%)	85 (11.8%)	0.541
Microbiology				
Negative blood culture	546 (65.6%)	68 (60.7%)	478 (66.4%)	0.240
S. aureus endocarditis	76 (9.1%)	15 (13.4%)	61 (8.5%)	0.093
Streptococci endocarditis	141 (16.9%)	23 (20.5%)	118 (16.4%)	0.277
Other	69 (8.3%)	10 (8.9%)	59 (8.2%)	0.793
Serum creatinine before surgery, μmol/L	82.25 ± 1.19	75.14 ± 2.35	82.04 ± 1.24	0.019
Aortic valve endocarditis, *n*	176 (21.2%)	16 (14.3%)	160 (22.2%)	0.056
Mitral valve endocarditis, *n*	320 (38.5%)	80 (71.4%)	240 (33.3%)	<0.001
Double valve endocarditis, *n*	320 (38.5%)	16 (14.3%)	304 (42.2%)	<0.001
Operation				
Isolated aortic valve replacement, *n*	176 (21.2%)	16 (14.3%)	160 (22.2%)	0.056
Isolated mitral valve surgery, *n*	320 (38.5%)	80 (71.4%)	240 (33.3%)	<0.001
Double valve operation, *n*	320 (38.5%)	16 (14.3%)	304 (42.2%)	<0.001
Bentall + MVR, *n*	16 (1.9%)	0	16 (1.9%)	
ECMO, *n*	3 (3.6%)	1 (0.9%)	2 (0.3%)	0.312

Vegetation length (12.98 ± 0.28 vs. 10.42 ± 0.17 mm, *P* < 0.001) in group with symptomatic neurological complications before surgery was significantly higher than that in group without symptomatic neurological complications before surgery (Table 1). Age (41.48 ± 0.64 vs. 43.54 ± 0.49 years, *P* = 0.028) and time between symptoms and surgery (1.95 ± 0.08 vs. 2.25 ± 0.06 months, *P* = 0.007) in group with symptomatic neurological complications before surgery were significantly less than those in group without symptomatic neurological complications before surgery (Table 1).

Mitral valve endocarditis and isolated mitral valve surgery (71.4% vs. 33.3%, *P* < 0.001) in group with symptomatic neurological complications before surgery were significantly higher than those in group without symptomatic neurological complications before surgery. Double valve endocarditis and double valve operation (14.3% vs. 42.2%, *P* < 0.001) in group with symptomatic neurological complications before surgery was significantly less than that in group without symptomatic neurological complications before surgery (Table 3).

Operative mortality (14.3% vs. 4.4%, *P* < 0.001), chest drainage (950.0 ± 58.3 vs. 583.3 ± 12.8 ml, *P* < 0.001), and fresh-frozen plasma (825.8 ± 69.0 vs. 611.6 ± 15.5 ml, *P* < 0.001) in group with symptomatic neurological complications before surgery were significantly higher than those in group without symptomatic neurological complications before surgery (Table 4).

**Table 4:** Operative data (*n* = 832).

Variable	Group with symptomatic neurological complications before surgery (*n* = 112)	Group without symptomatic neurological complications before surgery (*n* = 720)	*P* value
Operative death, *n*	16 (14.3%)	32 (4.4%)	<0.001
AKI, *n*	32 (28.6%)	240 (33.3%)	0.318
Aortic cross-clamp time	65.0 ± 2.89	90.16 ± 1.30	<0.001
Cardiopulmonary bypass time	116.43 ± 4.15	146.0 ± 1.96	<0.001
Intubation time, hours	44.79 ± 5.20	52.21 ± 2.32	0.235
ICU retention time, days	4.57 ± 0.26	5.00 ± 0.11	0.159
Hospitalized time postoperative, days	15.14 ± 0.57	20.11 ± 0.29	<0.001
Serum creatinine 24 h after surgery, μmol/L	82.57 ± 4.03	91.13 ± 1.65	0.056
Serum creatinine 48 h after surgery, μmol/L	99.43 ± 5.41	108.91 ± 2.69	0.185
Fluid balance on operation day, ml	−471.4 ± 68.8	−598.0 ± 28.0	0.096
Fluid balance on 1st day postoperative, ml	−500.0 ± 86.5	−620.0 ± 42.5	0.289
Fluid balance on 2nd day postoperative, ml	−585.7 ± 79.8	−542.2 ± 27.3	0.568
Chest drainage, ml	950.0 ± 58.3	583.3 ± 12.8	<0.001
Postoperative LVEDD, mm	46.29 ± 0.77	48.89 ± 0.25	<0.001
Postoperative LVEF, %	55.9 ± 0.8	59.0 ± 0.2	<0.001
Fresh-frozen plasma	825.8 ± 69.0	611.6 ± 15.5	<0.001
Packed red cells	2.86 ± 0.25	2.80 ± 0.13	0.864

Aortic cross-clamp time (65.0 ± 2.89 vs. 90.16 ± 1.30 min, *P* < 0.001), cardiopulmonary bypass time (116.43 ± 4.15 vs. 146.0 ± 1.96 min, *P* < 0.001), hospitalized time postoperative (15.14 ± 0.57 vs. 20.11 ± 0.29 days, *P* < 0.001), postoperative LVEDD (46.29 ± 0.77 vs. 48.89 ± 0.25 mm, *P* < 0.001), postoperative LVEF (55.9 ± 0.8% vs. 59.0 ± 0.2%, *P* < 0.001) in group with symptomatic neurological complications before surgery were significantly less than those in group without symptomatic neurological complications before surgery (Table 4).

The common early postoperative complications included acute renal injury (222/832, 26.7%), long-term intubation time >48 h (393/832, 47.2%), and multiorgan failure (86/832, 10.3%).

#### 3.3.1. Analysis of risk factors of symptomatic neurological complications before cardiac surgery in left-sided infective endocarditis (*n* = 1,760)

Univariate analysis of potential risk factors of symptomatic neurological complications before cardiac surgery in left-sided infective endocarditis showed that vegetation length (*P* < 0.001), aortic involvement (*P* < 0.001), and mitral involvement (*P* = 0.001) are associated with symptomatic neurological complications. When they were included in multivariate analysis models, multivariate analyses also showed that vegetation length (*P* < 0.001), aortic involvement (*P* = 0.004), and mitral involvement (*P* < 0.001) are associated with symptomatic neurological complications (Table 5).

**Table 5:** Analysis of risk factors of symptomatic neurological complications before surgery in left-sided infective endocarditis (*n* = 832).

Model	OR	95% CI	*P* value
Univariate analysis of risk factors of symptomatic neurological complications before surgery in infective endocarditis			
Time between symptoms and surgery	1.202	1.069–1.350	0.002
Preoperative aortic involvement	1.074	1.035–1.114	<0.001
Preoperative mitral involvement	0.937	0.906–0.968	<0.001
Serum creatinine before surgery	1.009	1.001–1.016	0.019
Multivariate analysis of risk factors of symptomatic neurological complications before surgery in infective endocarditis			
Time between symptoms and surgery	1.256	1.104–1.429	0.001
Preoperative aortic involvement	1.193	1.127–1.263	<0.001
Preoperative mitral involvement	0.915	0.881–0.950	<0.001
Serum creatinine before surgery	1.009	1.001–1.017	0.021

#### 3.3.2. Analysis of the significance of symptomatic neurological complications before surgery in left-sided infective endocarditis (*n* = 832)

Univariate and multivariate analysis of risk factors of in-hospital mortality following cardiac surgery, prolonged intubation time (intubation time >24 h), and 1-year mortality following cardiac surgery showed that symptomatic neurological complications before surgery is statistically significantly associated with in-hospital mortality following cardiac surgery (*P* < 0.001), prolonged intubation time (intubation time >24 h) (*P* < 0.05), and 1-year mortality following cardiac surgery (*P* < 0.001), respectively (Table 6).

**Table 6:** Analysis of the significance of symptomatic neurological complications before surgery in left-sided infective endocarditis (*n* = 832).

Model	OR	95% CI	*P* value
Univariate analysis of risk factors of in-hospital mortality following cardiac surgery (*n* = 48) in left-sided infective endocarditis			
Symptomatic neurological complications before surgery	3.583	1.895–6.775	<0.001
Multivariate analysis of risk factors of in-hospital mortality following cardiac surgery (*n* = 48) in left-sided infective endocarditis			
Symptomatic neurological complications before surgery	10.249	4.698–22.356	<0.001
Univariate analysis of risk factors of prolonged intubation time (intubation time >24 h, *n* = 352) following cardiac surgery in left-sided infective endocarditis			
Symptomatic neurological complications before surgery	2.000	1.294–3.092	0.002
Multivariate analysis of risk factors of prolonged intubation time (intubation time >24 h, *n* = 352) following cardiac surgery in left-sided infective endocarditis			
Symptomatic neurological complications before surgery	1.762	1.120–2.773	0.014
Univariate analysis of risk factors of 1-year mortality following cardiac surgery (*n* = 87) in left-sided infective endocarditis			
Symptomatic neurological complications before surgery	2.884	2.574–3.213	<0.001
Multivariate analysis of risk factors of 1-year mortality following cardiac surgery (*n* = 87) in left-sided infective endocarditis			
Symptomatic neurological complications before surgery	0.078	0.056–0.171	<0.001

### 3.4. Follow-up results

**Figure 2:**
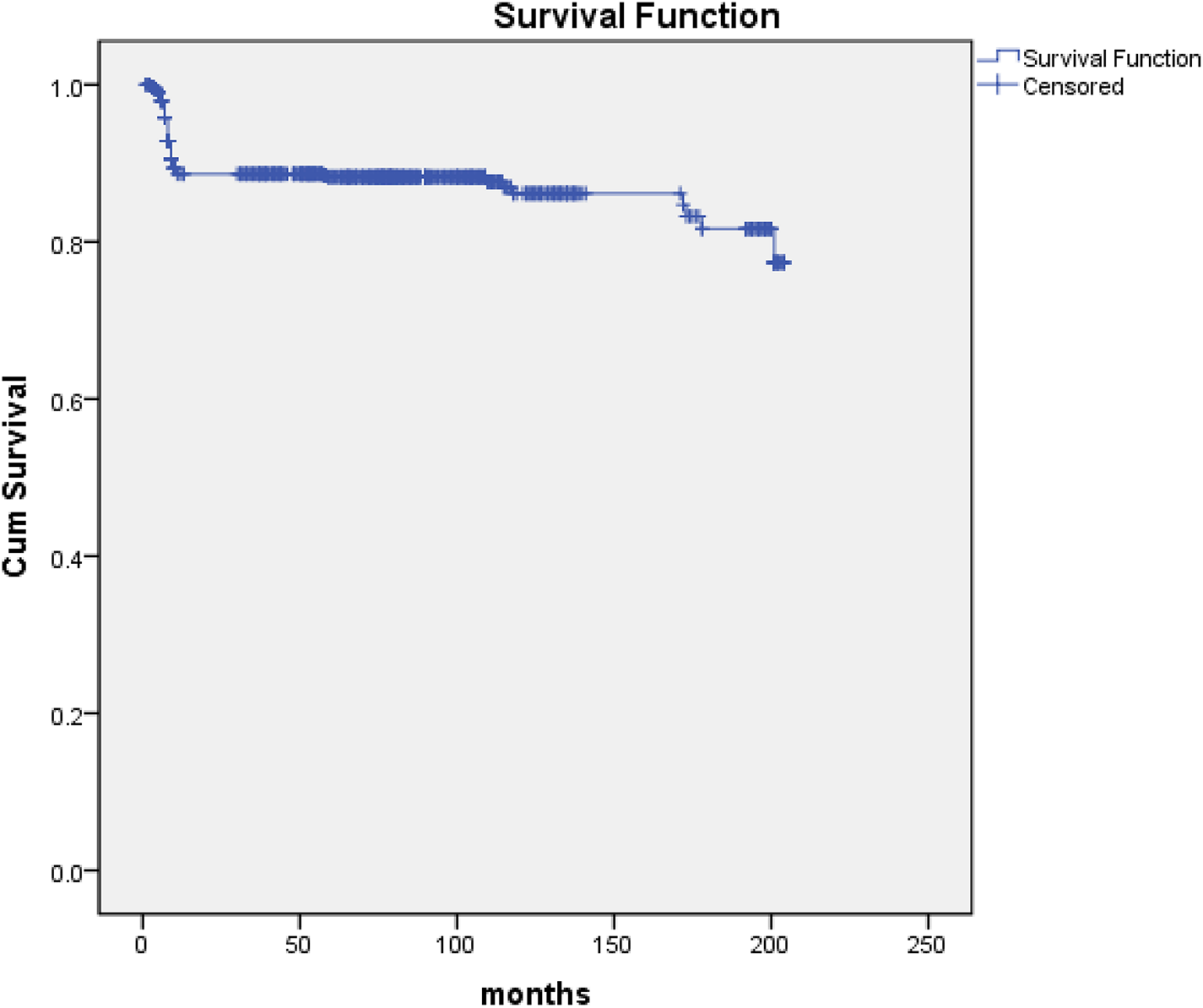
Kaplan-Meier curve for survival. The follow-up of 750 patients was completed (95.7%, 750/784). Eighty-seven deaths (11.6%, 87/750) occurred within 12 months after being discharged from our hospital because of recurrence of IE and cerebral hemorrhage.

Seven hundred eighty-four survivors were discharged from hospital and 750 patients were monitored to the end date of the study or a known date of death and the follow-up was 95.7% (750/784) completed. The mean duration of follow-up was 75.14 ± 1.80 months (range, 1–204). 87 deaths (87/750, 11.6%) occurred within 12 months after being discharged from our hospital because of recurrence of IE and cerebral hemorrhage. The latest data of follow-up showed that 639 survivors were in NYHA class I (639/663, 96.4%) and 24 in class II (24/663, 3.6%) (Figure 2).

## 4. Discussion

Infective endocarditis is an infectious disease associated with high morbidity and mortality. It is one of the most common life-threatening infections, occurring more frequently in older patients and those with prosthetic valves. Infective endocarditis patients with symptomatic neurological complications have a significantly higher risk of mortality than patients without symptomatic neurological complications. Surgery is an effective treatment in patients presenting with left-sided infective endocarditis and may be undertaken in patients with symptomatic neurological complications to prevent poorer prognosis.

Symptomatic neurological complications occur in 15%–30% of patients with left-sided infective endocarditis (10). The demographic characteristics of patients who develop infectious endocarditis (IE) have changed during the past few decades. Today in developed countries, patients tend to be older, their underlying diseases have changed, Staphylococcus aureus has emerged as a predominant causative organism, and there is an increasing incidence of health care-associated infections. In our study in China, 24.5% (432/1,760) of patients presented with symptomatic neurological complications, and operative mortality in group with symptomatic neurological complications before surgery was 14.3% (16/112), significantly higher than that (4.4%, 32/720) in group without symptomatic neurological complications before surgery (*P* < 0.001).

Common symptomatic neurological complications include transient ischemic attack, intracerebral hemorrhage, brain abscess, and toxic encephalopathy. However, silent cerebral embolisms also occur and may be associated with poorer prognosis. Anticoagulation during cardiopulmonary bypass may be particularly harmful for some patients. However, some studies have shown that neurological complications are not a contraindication for emergency surgery (11, 12).

Surgical treatment is particularly effective in selected IE patients presenting with neurological complications to prevent additional neurological sequelae and mortality. However, the decision for surgery remains controversial and should be decided carefully with a multidisciplinary team as patient prognosis may worsen with surgical intervention.

Presently, the decision of whether to intervene surgically after a stroke due to IE remains disputed. Recent studies indicate that early surgical treatment is a relatively safe and effective treatment option. Studies have also demonstrated that valvular surgery can be safely performed after silent cerebrovascular complications or transient ischemic attack. In the case of intracranial hemorrhage, it is suggested to delay surgery for at least 1 month due to a worse neurological prognosis. Preoperative cerebral imaging is a fundamental part of standard diagnostic methods, particularly in the case of neurological complications. The higher sensitivity of MRI could make it a good diagnostic tool; however, it is not as readily available, takes longer to perform, and has higher costs (13, 14).

### 4.1. Differences of neurological complications between aortic valve and mitral valve infective endocarditis

In our study, mitral valve endocarditis and isolated mitral valve surgery (71.4% vs. 33.3%, *P* < 0.001) in group with symptomatic neurological complications before surgery were significantly higher than those in group without symptomatic neurological complications before surgery, and double valve endocarditis and double valve operation (14.3% vs. 42.2%, *P* < 0.001) in group with symptomatic neurological complications before surgery was significantly less than those in group without symptomatic neurological complications before surgery (Table 3). As reported by previous studies, mitral valve endocarditis was associated with a greater stroke rate than aortic valve endocarditis, and strokes tended to occur early in the course of illness, particularly in mitral valve endocarditis (15, 16). Isolated mitral valve surgery takes less time than double valve operation, which can explain why aortic cross-clamp time (65.0 ± 2.89 vs. 90.16 ± 1.30 min, *P* < 0.001) and cardiopulmonary bypass time (116.43 ± 4.15 vs. 146.0 ± 1.96 min, *P* < 0.001) in group with symptomatic neurological complications before surgery were significantly less than those in group without symptomatic neurological complications before surgery in our study (Table 4).

In our study, univariate and multivariate analysis showed that symptomatic neurological complications before surgery is statistically significantly associated with in-hospital mortality following cardiac surgery, prolonged intubation time (intubation time >24 h), and 1-year mortality following cardiac surgery, respectively (Table 6). These results reflected that patients in group with symptomatic neurological complications before surgery were more serious, partially explaining why symptomatic neurological complications before surgery is associated with higher in-hospital mortality, prolonged intubation time, and 1-year mortality following cardiac surgery.

Patients with vegetations greater than 10 mm on echocardiography had a significantly higher risk of embolization and further neurological complications than patients with smaller vegetations, which suggests surgical intervention at this cutoff length (17–19). In our study, vegetation length (12.98 ± 0.28 vs. 10.42 ± 0.17 mm, *P* < 0.001) in group with symptomatic neurological complications before surgery were significantly higher than those in group without symptomatic neurological complications before surgery (Table 2), and univariate and multivariate analyses also showed that vegetation length (*P* < 0.001) are statistically significantly associated with symptomatic neurological complications before cardiac surgery in infective endocarditis (Table 5). Our study suggests that early cardiac surgery may prevent additional neurological complications in left-sided infective endocarditis.

### 4.2. Optimal time interval between neurological complications and surgery

The optimal time interval between symptomatic neurological complications and surgery is controversial, however, recent guidelines favor surgery in cases presenting with heart failure, uncontrolled infection, abscess formation, and those with high embolic risk. Left-sided infective endocarditis patients with symptomatic neurological symptoms scheduled for surgery should have a neurological evaluation by a neurologist, and brain imaging, either by CT or MRI, within days of the planned operation to visualize any strokes and to determine if an infarct is ischemic or hemorrhagic. Routine preoperative screening of asymptomatic patients, particularly those with high-risk vegetations, is justified. The standard recommendation is to delay surgery for 1–2 weeks in patients with non-hemorrhagic strokes, and for 3–4 weeks in patients with hemorrhagic strokes to reduce the risk of further intracranial bleeding during heart surgery. Patients with serious neurologic damage, unconscious patients, and those unable to follow simple commands should not be operated on until neurologic improvement has been demonstrated and potential for recovery has been established. For those with non-hemorrhagic embolic strokes, the main concerns are worsening the neurologic damage due to hemorrhagic conversion of the infarct and edema during the operation. The risk of worsening neurologic symptoms as a consequence of operation is time related, decreasing with increasing time from the initial neurologic event. If the patient is stable and risk of additional embolism is deemed to be low, delaying surgery for 1–2 weeks is probably beneficial, with repeat brain imaging before operation. The risk of worsening the stroke symptoms must be weighed against the indications for surgery and the risk of additional emboli during the waiting period. In conclusion, after a stroke, transient ischemic attack, or a silent cerebral embolism, surgery should be performed as soon as possible if coma is absent and cerebral hemorrhage has been excluded by cranial CT. In intracranial hemorrhage, surgery must be postponed for at least 1 month. Every patient should have a repeated head CT scan immediately before the operation to rule out a preoperative hemorrhagic transformation of a brain infarction (1, 20–23).

Symptomatic neurological complications before surgery are associated with increased in-hospital mortality following cardiac surgery and prolonged intubation time, some selected patients undergoing cardiac surgery recover satisfactorily. However, in our study, only 25.9% of patients (112/432) with symptomatic neurological complications are indicated for surgery at admission. How to diagnose and treat patients with symptomatic neurological complications effectively remains a great challenge for us.

### 4.3. Study limitations

Limitations of the present study include its retrospective design. There may be a selection bias because of the retrospective nature of the study. The nature of the study center (a tertiary referral center) may also induce a bias as patients with complicated forms of IE. Finally, we report the use of systematic CT-scan and not of MRI. This may have led to under diagnosis of neurological complications, especially small ischemic stroke and micro-bleeding. Well-designed research such as prospective cohort studies are needed and programs aiming at the reduction of in-hospital morbidity and mortality caused by left-sided infective endocarditis are encouraged.

## 5. Conclusions

Our study showed that symptomatic neurological complications before surgery are associated with higher in-hospital mortality following cardiac surgery and prolonged intubation time.

## Data Availability

The raw data supporting the conclusions of this article will be made available by the authors, without undue reservation.
