# Supplementary material for: Surgical treatment of left-sided infective endocarditis with symptomatic neurological complications before surgery in China
Source: Front Cardiovasc Med. 2023 Sep 6;10:1217148. doi: 10.3389/fcvm.2023.1217148 (PMC10510404; doi:10.3389/fcvm.2023.1217148)
Supplement: Supplementary file 1 [file Datasheet1.pdf]

Supplementary data

Variables were evaluated including gender (female/male), age, weight, time between symptoms and surgery, NYHA class, rheumatic heart disease, valvular heart disease, coronary heart disease, left ventricular end diastolic dimension, left ventricular ejection fractions, aortic insufficiency, mitral insufficiency, tricuspid insufficiency, serum creatinine, mean intubation time, ICU retention time, hospitalized time after surgery, postoperative chest drainage, fresh-frozen plasma, packed red cells, fluid balance on operation day, the first day following operation and the second day following operation, acute renal injury, multiorgan failure, long-term intubation, hepatic failure, respiratory failure, ventricular fibrillation, use of inotropic medication, blood lactate, extracorporeal membrane oxygenation (ECMO) requirement, and death.

Abbreviations

LVEDD =left ventricular end diastolic dimension； CT = computed tomography； CPB =cardiopulmonary bypass; ICU= intensive care unit; MOF=multiple organ failure ; ECMO= Extracorporeal Membrane Oxygenation; LVEF=left ventricular ejection fractions; AKI = acute renal injury; AKIN= acute kidney injury network； BMI (body mass index)=weight /($height^2$), (kg/m^2)； IE = infective endocarditis.
